# Steering Self‐Assembly of Three‐Dimensional Iptycenes on Au(111) by Tuning Molecule‐Surface Interactions

**DOI:** 10.1002/anie.202201044

**Published:** 2022-04-21

**Authors:** Lukas Grossmann, Eva Ringel, Atena Rastgoo‐Lahrood, Benjamin T. King, Johanna Rosen, Wolfgang M. Heckl, Dorina Opris, Jonas Björk, Markus Lackinger

**Affiliations:** ^1^ Deutsches Museum Museumsinsel 1 80538 Munich Germany; ^2^ Department of Physics Technische Universität München James-Franck-Str. 1 85748 Garching Germany; ^3^ Department of Chemistry University of Nevada Reno NV 89557-0216 USA; ^4^ Department of Physics, Chemistry and Biology Linköping University IFM, 581 83 Linköping Sweden; ^5^ Functional Polymers Empa Swiss Federal Laboratories for Materials Science and Technology 8600 Dübendorf Switzerland

**Keywords:** Crystal Engineering, Scanning Tunneling Microscopy, Self-Assembly, Surface-Passivation, Triptycene

## Abstract

Self‐assembly of three‐dimensional molecules is scarcely studied on surfaces. Their modes of adsorption can exhibit far greater variability compared to (nearly) planar molecules that adsorb mostly flat on surfaces. This additional degree of freedom can have decisive consequences for the expression of intermolecular binding motifs, hence the formation of supramolecular structures. The determining molecule‐surface interactions can be widely tuned, thereby providing a new powerful lever for crystal engineering in two dimensions. Here, we study the self‐assembly of triptycene derivatives with anthracene blades on Au(111) by Scanning Tunneling Microscopy, Near Edge X‐ray Absorption Fine Structure and Density Functional Theory. The impact of molecule‐surface interactions was experimentally tested by comparing pristine with iodine‐passivated Au(111) surfaces. Thereby, we observed a fundamental change of the adsorption mode that triggered self‐assembly of an entirely different structure.

## Graphical abstract

Passivation of Au(111) with an iodine monolayer provides effective means to steer the adsorption of three‐dimensional (f)antrip molecules. The stronger interaction on pristine Au(111) favours adsorption with two anthracene blades flat, promoting self‐assembly of a densely packed structure. By contrast, on passivated Au(111) f(antrip) adsorbs with its three anthracene blades edge‐on, giving rise to self‐assembly of a porous structure.

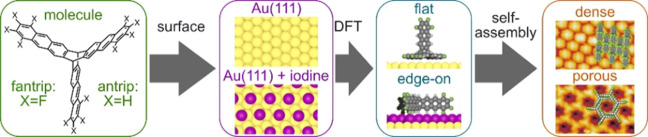

## Introduction

Most studies on surface‐supported supramolecular self‐assembly focus on (nearly) planar molecules,^[1]^ where a relevant share of the stabilization energy originates from molecule‐surface interactions. Their strength can even override molecule‐molecule interactions on strongly binding surfaces, with a pronounced if not decisive influence on resulting molecular structures. It is not astounding, therefore, that the balance of molecule‐surface and molecule‐molecule interactions remains the paradigm for the interpretation of structure formation on surfaces.^[2]^ In addition, planar molecules ideally meet the needs for prevalent characterization by Scanning Probe Microscopy.^[3]^ The routinely attained high resolution facilitates the direct determination of relative positions and orientations of individual molecules, from which intermolecular binding motifs are inferred. This real space crystallography has resulted in the direct determination of a myriad of surface structures that are intricate to assess by diffraction techniques.^[4]^

Currently, studies of three‐dimensional molecules with highly non‐planar structures on surfaces are scarce, with fullerenes constituting a notable exception.^[5]^ Yet, for C_60_ the adsorption configuration does not critically influence intermolecular binding due to its high symmetry and spherical shape. In this respect, less symmetric three‐dimensional molecules can exhibit vastly different modes of adsorption with concomitant consequences for expressing distinct intermolecular binding motifs. Consequently, tuning the strength of molecule‐surface interactions provides effective means to steer the adsorption geometry of three‐dimensional molecules, hence offering a new, currently underexplored level of control for structure formation.

Iptycenes are an intriguing class of highly stable compounds with three‐dimensional aromatic structures. In these formal barrelene derivatives, arenes of variable size and functionalization are fused by bridged bicyclo‐octatriene cores. First iptycenes were proposed by Clar in 1932,^[6]^ and triptycene (tribenzobicyclo[2.2.2]octatriene) as the archetypical representative was synthesized less than a decade later.^[7]^ Iptycenes can serve as core units for the synthesis of fascinating macromolecules.^[8]^ Moreover, their noncompliant three‐dimensional structures can favorably alter the packing in polymers, either as an integral part of the macromolecule's backbone or as additives, giving rise to advanced mechanical and optical properties.^[9]^ Importantly, iptycenes are promising candidates for a range of applications in photo‐ and materials chemistry.^[10]^

**Figure 1:**
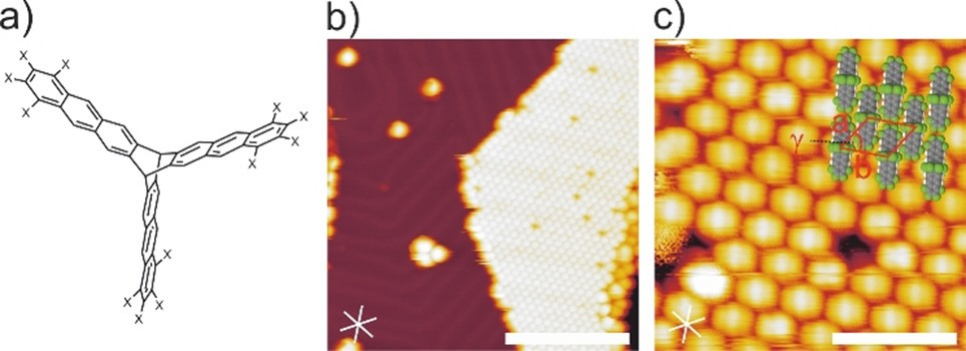
a) Chemical structures of fantrip (X=F) and antrip (X=H); b) Overview and c) close‐up STM images of fantrip on pristine Au(111); crosses indicate high symmetry directions of the substrate; The overlay in (c) shows the proposed adsorption geometry of individual fantrip molecules and their dense packing. (tunneling parameters and scale bars: b) −2.80 V, 3 pA, 20 nm; c) −2.80 V, 3 pA, 4 nm).

Here, we study the self‐assembly of three‐fold anthracene‐triptycene derivatives (cf. Figure 1a for structures). Extended anthracene blades enhance the stability of supramolecular structures with an impact on both molecule‐molecule and molecule‐surface interactions. The fluorinated (fantrip) and non‐fluorinated (antrip) analogs were devised as monomers to synthesize 2D polymers by a topochemical photopolymerization either in monolayers or layered bulk crystals.^[11]^ Therefore, self‐assembly into a reactive packing, where all anthracene blades of each monomer are face‐to‐face stacked is essential to facilitate their lateral polymerization by intermolecular [4+4] photocycloadditions.^[12]^ Accordingly, crystal engineering has become an integral part of photochemical 2D polymer synthesis. We used graphite substrates in our previous study of fantrip's on‐surface photopolymerization.^[11d]^ Yet, even on this weakly interacting substrate, alkane‐passivation has proven necessary to obtain the reactive packing. For the advancement and fundamental understanding of on‐surface photopolymerization, it is highly desirable to extend the portfolio of applicable surfaces also to metals. Surprisingly, even on comparatively weakly interacting Au(111) surfaces, alkane‐passivation turned out insufficient to prevent self‐assembly of a structure that is determined by molecule‐surface interactions.^[11d]^ Here, we present a comprehensive study of fantrip and antrip adsorption and self‐assembly on pristine Au(111). Furthermore, we explore the effect of strong surface‐passivation by an iodine monolayer. Structure determination rests on a combination of Scanning Tunneling Microscopy (STM) and Near Edge X‐ray Absorption Fine Structure (NEXAFS) analysis. Further insights into the energetics are gained by Density Functional Theory (DFT) calculations.

## Results and Discussion

**Figure 2:**
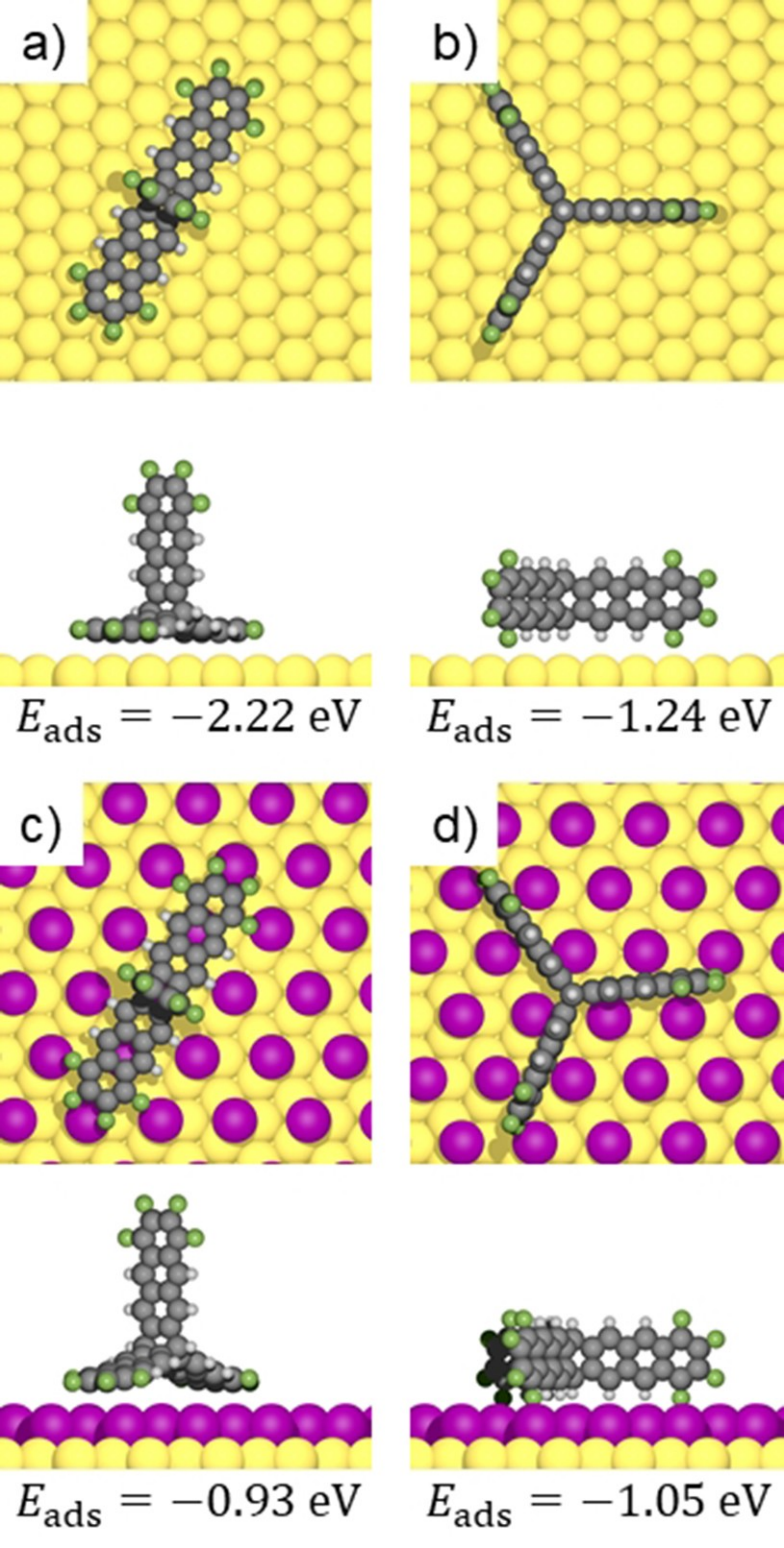
DFT optimized adsorption geometries for single fantrip molecules on a)/b) pristine and c)/d) iodine‐passivated Au(111). Subfigures (a)/(c) depict adsorption geometries with two anthracene blades flat, while in (b)/(d) all anthracene blades adsorb edge‐on. The corresponding side views are shown below each panel, and respective adsorption energies are indicated below (see Supporting Information for a full comparison of different calculated adsorption sites for each adsorption geometry on both passivated and pristine Au(111)).

First, we studied fantrip self‐assembly on pristine Au(111). The representative STM images in Figures 1b and c show the exclusively observed nearly hexagonal phase with lattice parameters *a*=1.22±0.06 nm, *b*=1.45±0.04 nm and *γ*=58°±2°. It contains one single oval protrusion per unit cell that is, according to its extent and spacing, assigned to a single fantrip molecule. We propose an adsorption geometry as illustrated by the overlay in Figure 1c, where each fantrip adsorbs with two anthracene blades flat on Au(111) while the third stands upright. This adsorption geometry is also compatible with the observed packing and becomes energetically favorable through strong anthracene‐Au(111) interactions, surpassing the energy cost for deformation of fantrip from its ideal three‐fold symmetry. This intuitive rationale is substantiated by the DFT calculations summarized in Figure 2. The proposed structure with two anthracene blades adsorbed is associated with a high binding energy of −2.22 eV, while hypothetical adsorption of nearly undeformed fantrip with its three‐fold axis perpendicular to the surface and all three anthracene blades adsorbed edge‐on is significantly weaker with an adsorption energy of −1.24 eV (Figure 2b). Notably, these energies refer to optimized isolated fantrip molecules in vacuum, hence already include the deformation energy. The associated energy cost of 0.36 eV for deforming fantrip to the geometry shown in Figure 2a is non‐negligible, but is easily surpassed by the significantly stronger interaction with Au(111). The dense packing as inferred from STM images is corroborated by DFT calculations of free‐standing monolayers (Supporting Information), which also indicate additional stabilization by intermolecular C−F⋅⋅⋅H−C hydrogen bonds between the flat adsorbing anthracene blades.

**Figure 3:**
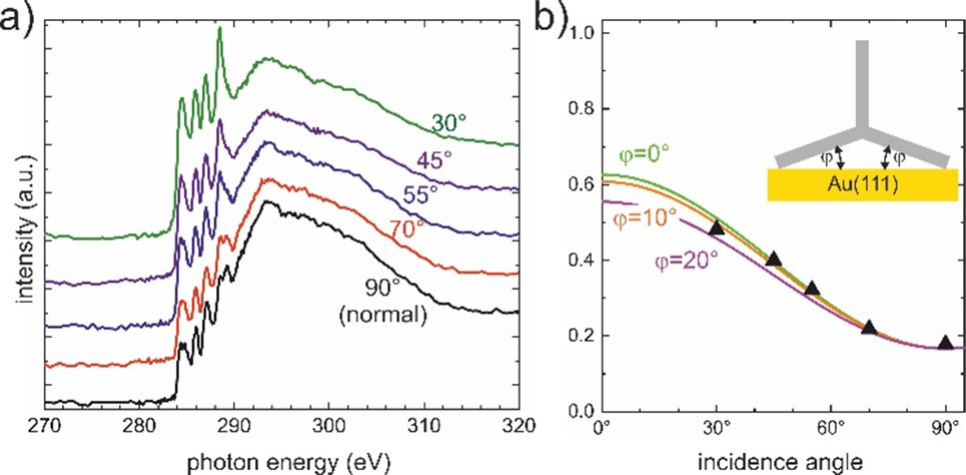
NEXAFS of fantrip on pristine Au(111). a) Carbon K‐edge spectra acquired for incidence angles between 30° and 90° (normal incidence); b) intensity plots derived from a); data points are shown as filled triangles; the solid lines correspond to theoretical intensity plots computed for different dihedral angles *ϕ* between Au(111) and the two flat adsorbing anthracene blades as illustrated in the insert.

Even though the combination of STM and DFT already provides strong evidence for the proposed adsorption geometry, NEXAFS experiments were carried out for a more direct and quantitative structure determination. Carbon K‐edge spectra acquired for X‐ray incidence angles between 30° and 90° are summarized in Figure 3a. Four resonances are clearly resolved that arise from a combination of initial and final state effects. Fluorine substitution gives rise to large chemical shifts in C 1s binding energies,^[13]^ and transitions from both unsubstituted and fluorine‐substituted carbon atoms into unoccupied π_1_* and π_2_* molecular orbitals explain the occurrence of four resonances. From these spectra, the intensity versus incidence angle plot shown in Figure 3b was derived. The highest intensity was observed for the smallest, i.e. most grazing incidence angle. It declines with increasing incidence angle, but remains at a relatively large base value for normal incidence. This already indicates predominantly but not entirely flat adsorption of the aromatic moieties. For modeling, we presumed the anthracene blades as planar and rigid, and further assumed additivity of their contributions. In the proposed mirror symmetric adsorption geometry, two anthracene blades form a similar dihedral angle *ϕ* with the Au(111) surface, whereas the third anthracene blade stands upright (cf. insert in Figure 3b). We then computed theoretical intensity plots with *ϕ* as a single parameter that fully defines the adsorption geometry, whereby a satisfying agreement was attained for *ϕ*=10°. Accordingly, NEXAFS corroborates the adsorption geometry inferred from combining STM with DFT, and indicates almost flat adsorption of two anthracene blades on Au(111) under the assumption that the third anthracene blade statically stands upright.

All results for fantrip on pristine Au(111) consistently indicate self‐assembly of a structure that is determined by strong molecule‐surface interactions. The imposed adsorption geometry hampers face‐to‐face stacking of the anthracene blades as the strongest possible intermolecular binding motif. The intermolecular hydrogen bonds are, hence, interpreted as a consequence rather than a driver of the densely packed fantrip self‐assembly. Accordingly, overcoming the predominant molecule‐surface interactions on pristine Au(111) bears the potential to alter the mode of adsorption, and as a result the type of intermolecular binding, with profound consequences for the supramolecular arrangement. An attempt in this direction was previously made by passivating Au(111) with a hexacosane monolayer.^[11d]^ Yet, this alkane‐passivation proved insufficient for affecting fantrip's mode of adsorption, indicating a remarkably long range of the anthracene‐Au(111) interactions.

Alternatively, we here propose stronger passivation of Au(111) by a monolayer of chemisorbed iodine that forms a commensurate *p*√3×√3R30° superstructure (I−Au(111)).^[14]^ Previously, we used intercalation of an iodine monolayer between covalent networks and the metal surfaces on which they were grown for their post‐synthetic decoupling.^[15]^ From X‐ray standing wave experiments, we inferred an increase of the average adsorption height of covalent triazine‐phenylene networks from 0.31 nm on pristine Ag(111) to 0.59 nm upon iodine intercalation.^[16]^ This implies highly efficient decoupling by the iodine monolayer, which for fantrip becomes readily apparent in the DFT calculations in Figure 2. In comparison to pristine Au(111), fantrip adsorption not only becomes generally weaker on I−Au(111) as similarly inferred for hexadecylamine on chlorine and iodine passivated copper surfaces,^[17]^ but even the energetic ordering of the two competing structures is reversed. Adsorption of fantrip with its three‐fold axis perpendicular to the surface is associated with an adsorption energy of −1.05 eV (Figure 2d). Notably, this value exceeds the adsorption energy of −0.93 eV for fantrip adsorption with two anthracene blades flat (Figure 2c), i.e. the clearly preferred configuration on pristine Au(111). We quantify two of the underlying reasons: (1) The fantrip deformation energy for flat anthracene adsorption of +0.18 eV is smaller on I−Au(111) than on pristine Au(111), but significantly larger than the negligible deformation energy of 5 meV for edge‐on adsorption with an additional contribution of 3 meV for rearrangements in the iodine layer. (2) For edge‐on adsorption fantrip preferentially adsorbs within the troughs between the iodine rows (Figure 2d). This facilitates closer proximity to Au(111), which results in an energetic advantage of about 0.13 eV for the direct interactions with the metal surface as compared to the adsorption geometry with two anthracenes flat (Supporting Information). In addition, a lower adsorption height also furnishes an increase of van‐der‐Waals interactions with the iodine monolayer as all interatomic distances become smaller. According to a Bader charge analysis, the iodine atoms in the unperturbed monolayer acquire a modest negative charge of −0.078 e. Upon fantrip adsorption, only a minor component of this negative charge is shifted away underneath the electronegative fluorine‐substituents toward the center of the molecule as unveiled by the respective charge difference plot (Supporting Information). Yet, we do not find indications for a specific interaction between the halogens, i.e. iodine and fluorine. This leads us to the conclusion that the main reason for preferred adsorption in the iodine troughs is indeed of steric nature: it simply features closer proximity to and stronger interaction with both the Au(111) surface and the iodine monolayer. Moreover, a comparison of fantrip's calculated partial density of electronic states shows hardly any changes upon adsorption on I−Au(111) (Supporting Information). We interpret this as a clear signature of physisorption, in line with the modest charge transfer of −0.14 e from the surface to the molecule.

**Figure 4:**
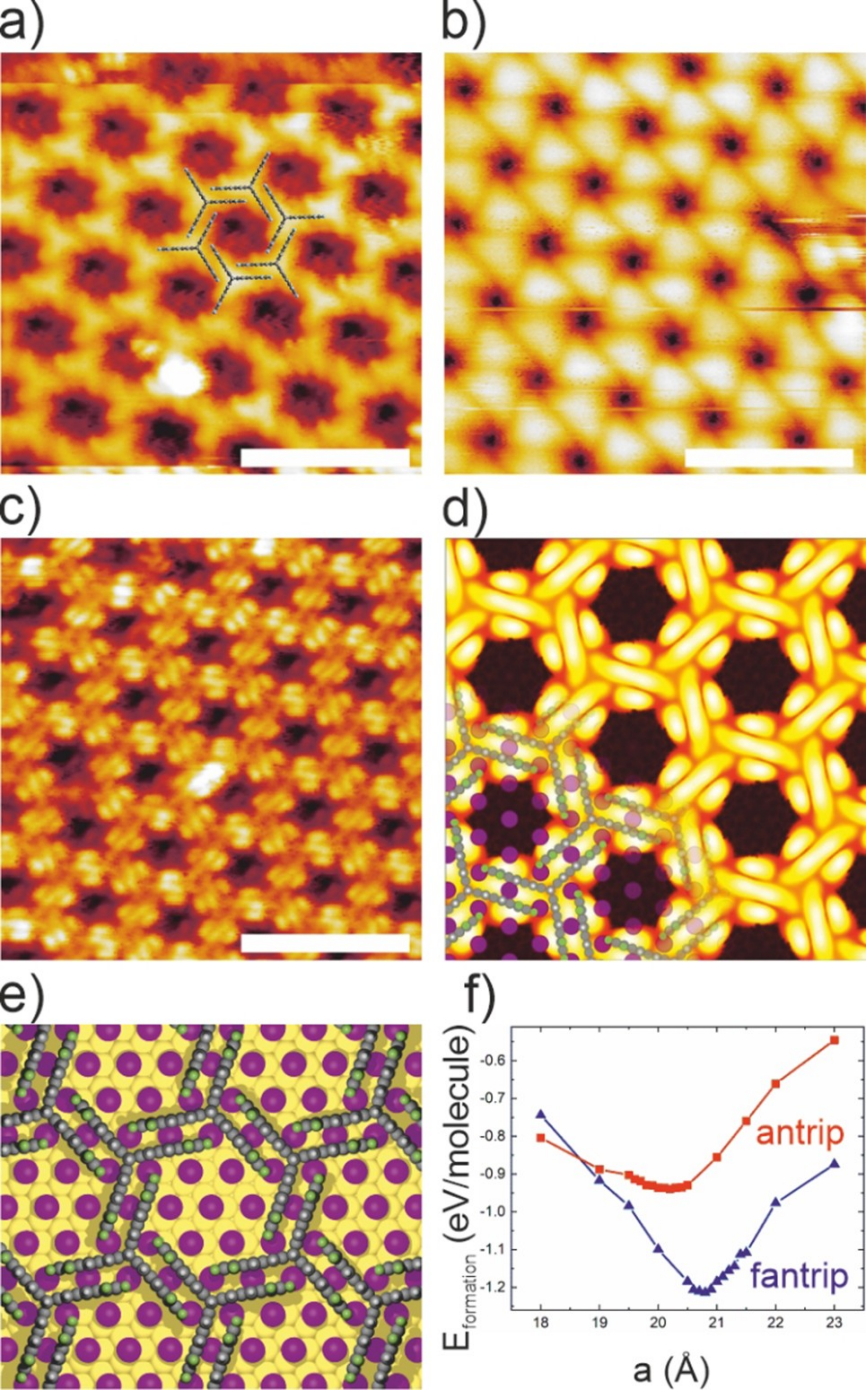
a)–c) STM images of the hexagonal fantrip structure on I−Au(111) showing the typically observed contrast variations. Fantrip's appearance in a) as trigonal stars unveils the packing's organizational chirality as illustrated by the overlay. While in (b) individual fantrip molecules appear as triangles without internal structure, in (c) π‐electron clouds protruding out of upright anthracene blades give rise to a pronounced contrast as similarly observed on alkane‐passivated graphite.^[11d]^ This contrast is in perfect agreement with the STM image simulation in (d) that is based on the DFT calculated structure of the full monolayer adsorbed on I−Au(111) shown in (e); For the simulation occupied electronic states up to −2.0 eV below the Fermi energy were considered. f) DFT calculations of the intermolecular binding energies per molecule in the free‐standing face‐to‐face stacked porous structure as a function of lattice parameter in a hexagonally constrained unit cell. Full triangles and squares correspond to fantrip and antrip, respectively and the lines serve as guides to the eye. (tunneling parameters: a) 2.5 V; 4 pA; b)+3.00 V, 4 pA; c) −2.20 V; 4 pA; all scale bars 4 nm).

This initial theoretical assessment is directly corroborated by experiments. Upon deposition of fantrip on I−Au(111), STM unveils a new hexagonal structure with an enlarged lattice parameter of 2.01±0.05 nm. Moreover, we routinely observe different STM contrasts as summarized in Figure 4. The three‐fold symmetry of individual fantrip molecules is particularly apparent in Figure 4a, clearly indicating adsorption with its anthracene blades edge‐on. The overlay also unveils the handedness of the packing's organizational chirality. The detailed experimentally observed contrast in Figure 4c offers a scarce side view on the aromatic anthracene blades, showing the protruding π‐electron clouds. Moreover, it can be reproduced with high fidelity by STM image simulation shown in Figure 4d. Yet, the other observed contrasts could not be matched with simulations for a range of bias voltages (Supporting Information), hence are attributed to dominating contributions of the STM tip. The proposed anthracene edge‐on adsorption geometry on I−Au(111) is corroborated by NEXAFS experiments (Supporting Information).

Yet, observation of the hexagonal monolayer does not directly confirm the DFT predicted energetic preference of edge‐on adsorption for individual molecules, because the observed hexagonal packing gains a substantial stabilization of −1.24 eV per molecule from face‐to‐face stacking of all anthracene blades (Figure 4f) to be compared with −0.34 eV per molecule for the packing in Figure 1c (Supporting Information). On alkane‐passivated graphite, these molecule‐molecule interactions are indispensable for the stabilization of the hexagonal structure.^[11d]^ To further explore whether the diminished molecule‐surface interactions on I−Au(111) by themselves already account for the change of adsorption mode, single molecules must be studied. Therefore, we deposit fantrip onto I−Au(111) held at ≈80 K to hamper molecular mobility and self‐assembly, resulting in small aggregates that at least partly corroborate the DFT calculations (Supporting Information).

STM also unveils that fantrip adopts a commensurate 4×4 superstructure on I−Au(111) with respect to the iodine lattice (Supporting Information). The corresponding lattice parameter of 2.00 nm is ≈4 % smaller than the 2.08 nm obtained for free‐standing fantrip monolayers from DFT (Figure 4f). By contrast, the larger experimental lattice parameter of 2.05 nm on alkane‐passivated graphite and the absence of a unique azimuthal orientation indicate a weaker surface influence.^[11d]^ Based on the large site variability of fantrip adsorption energies on I−Au(111) (Supporting Information), we propose that the energy cost associated with the compressive strain in the 4×4 superstructure is compensated by optimization of fantrip's adsorption sites. Remarkably, both non‐equivalent fantrip molecules in the unit cell can simultaneously adopt the markedly preferred adsorption geometry depicted in Figure 2d. This is corroborated by DFT calculations of the full monolayer on I−Au(111) shown in Figure 4e. Thereby, the binding energy per molecule in the adsorbed monolayer amounts to −2.15 eV, which is astonishingly close to the sum of the individually calculated adsorption energy of single molecules (−1.05 eV, Figure 2d) and intermolecular binding energy in free‐standing monolayers (−1.24 eV, Figure 4f). A further remarkable feature of fantrip self‐assembly on I−Au(111) is the ability to coherently overgrow monoatomic steps (Supporting Information). STM line‐profiles indicate that the required height offset is directly accommodated at the step‐edge.

To shed more light on the influence of the anthracene's peripheral fluorination, we also studied its non‐fluorinated analog antrip (Figure 1a). STM data acquired after deposition of antrip onto pristine and iodine‐passivated Au(111) are summarized in the Supporting Information. The observed antrip structures are essentially similar to those of fantrip, i.e. adsorption with two anthracene blades flat on pristine Au(111) and self‐assembly of the face‐to‐face stacked hexagonal structure on I−Au(111). Only the molecular packing on pristine Au(111) exhibits notable differences for antrip owing to combined effects of molecule‐molecule and molecule‐surface interactions (Supporting Information). Despite the absence of qualitative differences, it is nevertheless instructive to further explore details.

Therefore, we assessed the energetics of intermolecular interactions in free‐standing face‐to‐face stacked monolayers by DFT calculations. The graph in Figure 4f depicts the intermolecular binding energy per molecule in hexagonally constrained fantrip/antrip monolayers as a function of lattice parameter. Important differences are evident. For fantrip the equilibrium distance of 2.08 nm (corresponding to the energy minimum) is slightly larger than for antrip (2.02 nm), but the binding energy is notably enhanced for fantrip (−1.24 eV) as compared to antrip (−0.94 eV). Moreover, the energy minimum is shallower for antrip, i.e. the energy cost associated with a small increase from the equilibrium distance is smaller. All these differences can consistently be explained by fantrip's peripheral fluorine‐substituents. The larger atomic radius of fluorine accounts for the increase in distance between the face‐to‐face stacked anthracenes and lattice parameter, respectively. But the peripheral fluorine substituents also give rise to a sizable negative electrostatic potential at the periphery of the anthracene blades (Supporting Information). Accordingly, the attractive electrostatic interactions with the positive potential at the hydrogen substituents in the antiparallel stacking of the fluorinated anthracene blades enhance the binding energy, and also account for a more rapid decay with increasing distance. Noteworthy, charge rearrangements in the monolayer packing also indicate additional stabilization by π‐π interactions between the anthracene blades in the more perpendicular T‐shaped configuration. By contrast, the anthracene‐anthracene interaction between antrip molecules lacks this pronounced electrostatic interaction and is merely of van‐der‐Waals type.

## Conclusion

Passivation by an iodine monolayer turned out to be a viable means for completely altering the mode of adsorption of three‐dimensional anthracene‐triptycene derivatives on Au(111). On pristine Au(111), adsorption of both fantrip and antrip is governed by strong molecule‐surface interactions that tend to maximize the area of contact, resulting in adsorption with two anthracene blades flat. Consequently, self‐assembly mostly follows the principle of close packing. Yet, for fantrip intermolecular hydrogen bonds afford an evenly distributed structure, while the antrip monolayer exhibit an imprint of molecule‐surface interactions.

Iodine passivation drastically alters the adsorption geometry, presumably at the single molecule level, rendering the edge‐on adsorption of anthracenes energetically favorable. Once the flat adsorption of the anthracene blades has been overcome, they undergo face‐to‐face stacking as the strongest and most specific intermolecular binding motif that is enhanced for fantrip by attractive electrostatic interactions. Consequently, we directly observe the hexagonal porous structure in which this interaction is optimized. The structure with face‐to‐face stacking of all anthracenes is particularly desirable owing to its defined porosity, but also as reactive packing for the photochemical synthesis of 2D polymers. We hope our work on iptycenes stimulates future research on this highly intriguing class of compounds with three‐dimensional aromatic structures.

## Conflict of interest

The authors declare no conflict of interest.

1

## Supporting information

As a service to our authors and readers, this journal provides supporting information supplied by the authors. Such materials are peer reviewed and may be re‐organized for online delivery, but are not copy‐edited or typeset. Technical support issues arising from supporting information (other than missing files) should be addressed to the authors.

Supporting Information

## Data Availability

The data that support the findings of this study are available from the corresponding author upon reasonable request.
