# Supplementary material for: Steering Self‐Assembly of Three‐Dimensional Iptycenes on Au(111) by Tuning Molecule‐Surface Interactions
Source: Angew Chem Int Ed Engl. 2022 Apr 21;61(25):e202201044. doi: 10.1002/anie.202201044 (PMC9325367; doi:10.1002/anie.202201044)
Supplement: Supplementary file 1 — Supporting Information [file ANIE-61-0-s001.pdf]

Angewandte Chemie
Eine Zeitschrift der Gesellschaft Deutscher Chemiker

Supporting Information

Steering Self-Assembly of Three-Dimensional Iptycenes on Au(111) by Tuning Molecule-Surface Interactions

L. Grossmann, E. Ringel, A. Rastgoo-Lahrood, B. T. King, J. Rosen, W. M. Heckl, D. Opris, J. Björk, M. Lackinger**

# – Supporting Information –

Table of Contents

1 Materials and Methods 2
1.1 Sample preparation and STM characterization 2
1.2 NEXAFS experiments 2
1.3 DFT calculations 3
2 NEXAFS of fantrip on iodine-passivated Au(111) 4
3 Additional STM data 4
3.1 Epitaxial relation of fantrip and antrip on I-Au(111) 4
3.2 Fantrip on iodine-passivated Au(111) 5
3.3 Antrip on pristine Au(111) 6
3.4 Antrip on iodine-passivated Au(111) 6
4 Additional DFT results 7
4.1 Free-standing monolayers 7
4.2 Single fantrip molecules on pristine Au(111) 9
4.3 Single fantrip molecules on iodine-passivated Au(111) 10
4.4 STM image simulations 13
5 Synthesis of fantrip and antrip 14
6 References 14

1 Materials and Methods

1.1 Sample preparation and STM characterization

All STM experiments were carried out in ultra-high vacuum (UHV) at a base pressure of ~3×10^{-10} mbar. Au(111) single crystals were used as substrates and cleaned in-situ by the usual cycles of Ar^+-ion sputtering (0.5…1.0 keV, 10 min) and annealing (450 °C - 500 °C, 10 min). The surfaces were checked by STM imaging, where emergence of an unperturbed herringbone reconstruction indicated their cleanliness.

Fantrip and antrip were deposited from home-built molecular evaporators using crucible temperatures of 240 °C (fantrip) and 250 °C (antrip) and deposition times of 15…45 min. Iodine-passivated Au(111) (I-Au(111)) samples were prepared by exposing freshly prepared Au(111) substrates to I_2 vapour (~9×10^{-6} mbar) for ~20 min in a separate preparation chamber to avoid cross-contamination of the main chamber. Thereby, holding the Au(111) surface at an elevated temperature of ~200 °C and cooling down in the I_2 atmosphere aided in obtaining a completely closed iodine monolayer devoid of vacancies. Alternatively, samples were annealed to ~150°C after the iodination to form the monolayer.

We used a home-built low-temperature STM operated by a BP4 control system (Nanonis) for sample characterization. STM imaging was carried out at ~90 K using electrochemically etched tungsten tips that were in-situ conditioned by Ar^+-ion sputtering. Stated tunneling voltages refer to the sample. STM images were processed by levelling and mean filtering.

1.2 NEXAFS experiments

NEXAFS experiments were carried out at BESSY II synchrotron (Helmholtz-Zentrum Berlin) at the HE-SGM beamline. Fantrip on pristine Au(111) samples were prepared as outlined above by in-situ deposition. Yet, iodination of Au(111) samples was not possible at the beamline. Hence, we prepared I-Au(111) samples in advance in our home laboratory. These samples were characterized by STM, stored under vacuum and swiftly transferred through atmosphere to UHV at the synchrotron. For these experiments we used Au(111) films on mica (Georg Albert PVD-Beschichtungen) as substrates.

Carbon K-edge NEXAFS spectra were acquired for incidence angles of 30°, 45°, 55°, 70° and 90° (=normal incidence) with respect to the surface plane. A home-built double channel plate detector was used in partial electron yield (PEY) mode, i.e. with a counter voltage of -150 V applied. Photon energies were calibrated by means of an internal carbon reference. Spectra of clean Au(111) were used for both background and photon flux correction.

Intensities of the C 1s → π^* resonances were determined as the peak maximum values. Theoretical curves were calculated for the (111) surfaces by using the standard equation for three-fold symmetric surfaces,[1] and by considering the beamline-specific degree of linear polarization of P=0.92.

1.3 DFT calculations

Periodic Density Functional Theory (DFT) calculations were performed with the VASP code,[2] using the projector-augmented wave method to describe ion-core interaction.[3] Exchange-correlation were described by the van der Waals density functional (vdW-DF),[4] in the recent form by Hamada,[5] denoted by rev-vdW-DF2, which has been shown to accurately describe molecular adsorption in a variety of systems.[5-6] The Au(111) surface was represented by a slab of four layers. We used a $(5\sqrt{3} \times 5\sqrt{3})\mathrm{R}30°$ surface unit cell (with respect to the primitive Au(111) unit cell) for all calculations of the adsorption of isolated fantrip molecules on both pristine and iodine-passivated Au(111). The iodine monolayer was modelled as $p(\sqrt{3} \times \sqrt{3})R30°$ superstructure with iodine atoms adsorbed in three-fold hcp-sites in accord with literature.[7] For all adsorption configurations, we first performed calculations with a Γ-point only *k*-point sampling (Figures S7 - S10), and for the most stable adsorption configurations calculations with a 2×2 *k*-point sampling to ensure full convergence of adsorption energies (Figure 2 of main manuscript). The *k*-point sampling shifted the absolute values of adsorption energies by less than 30 meV for each case. For the calculations of the adsorbed supramolecular hexagonal structure of fantrip molecules on I-Au(111) a $(5\sqrt{3} \times 5\sqrt{3})\mathrm{R}30°$ surface unit cell was used together with a 2×2 *k*-point sampling. Furthermore, a 400 eV kinetic energy cutoff was applied for the calculations of fantrip adsorption on Au(111) and I-Au(111). For calculations of free-standing fantrip and antrip monolayers, a 450 eV kinetic energy cutoff was used to ensure convergence when comparing calculations that used different unit cell sizes. Structural optimizations were performed on all atoms – except the bottom two Au layers of the Au slab, which were kept frozen – until the residual forces were smaller than 0.01 eV/Å. STM image simulations were performed within the framework of the Tersoff-Hamann approximation,[8] using the implementation by Lorente and Persson.[9]

2 NEXAFS of fantrip on iodine-passivated Au(111)

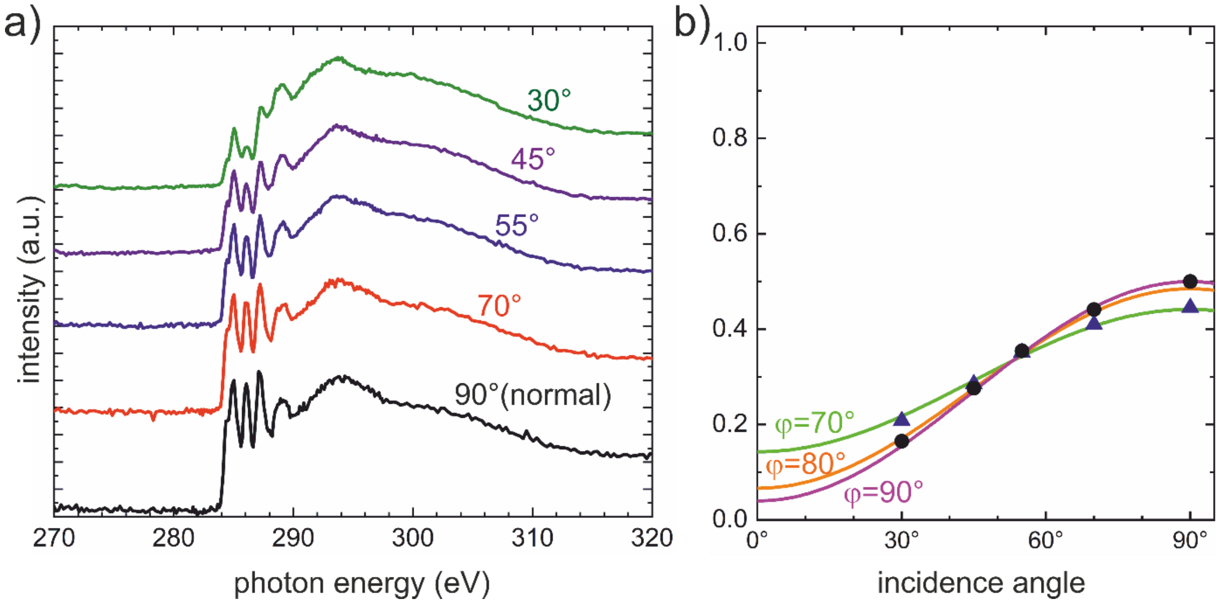

Fig. S1 NEXAFS of fantrip on I-Au(111). **a)** Carbon K-edge spectra acquired for incidence angles between 30° and 90° (normal incidence); **b)** intensity plots derived from a); data points are shown as filled symbols; Filled triangles correspond to intensities from the first resonance at 285.0 eV, whereas filled circles correspond to the second resonance at 286.1 eV. The solid lines represent theoretical intensity plots computed for different dihedral angles φ between Au(111) and anthracene blades, where φ=90° corresponds to a fully upright, i.e. edge-on orientation. Accordingly, edge-on adsorption of the anthracene blades is corroborated.

3 Additional STM data

3.1 Epitaxial relation of fantrip and antrip on I-Au(111)

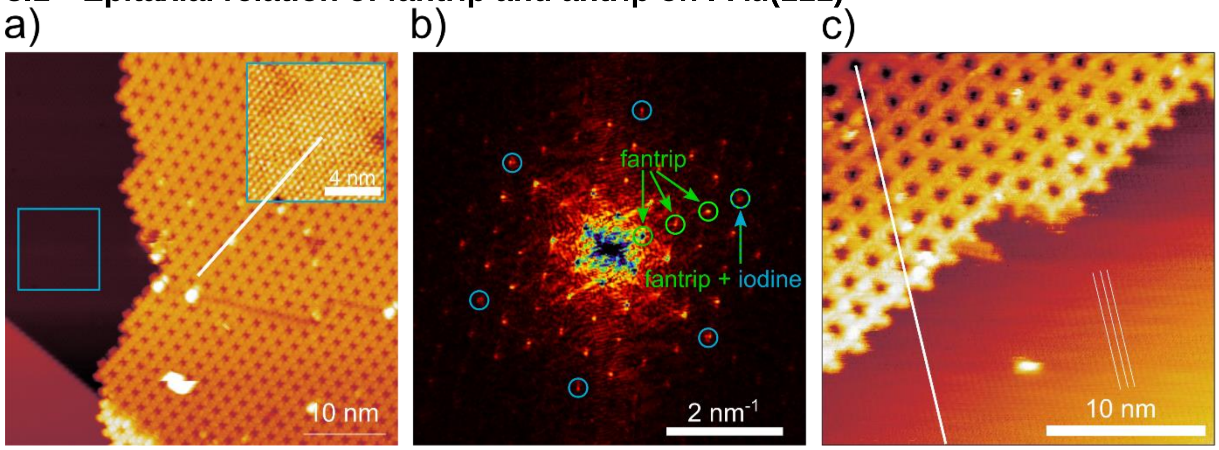

Fig. S2 Epitaxial relation of hexagonal **a) / b)** fantrip and **c)** antrip monolayers on I-Au(111) surfaces. Both fantrip and antrip adopt a commensurate 4×4 superstructure, despite differences in their optimized lattice parameters of free-standing monolayers (cf. Figure 4f of main manuscript). a) Shows a large scale image of a fantrip monolayer. The similar orientation of the underlying iodine lattice (highlighted by the white line) was inferred from atomically resolved STM images of the iodine monolayer (c.f. insert, the image was acquired at the position indicated by the blue square). The corresponding FFT in b) shows peaks from both fantrip and iodine structures. Coincidence of fantrip's (40) with iodine's (10) peak directly confirms the 4×4 superstructure. **c)** STM image simultaneously showing both an antrip monolayer and the underlying iodine monolayer. Their mutual alignment and the measured antrip lattice parameter of 1.95 ± 0.10 nm likewise corroborate a 4×4 superstructure. (tunneling parameters and scale bars: a) -2.20 V, 4 pA, 20 nm; c) +1.80 V, 27 pA, 10 nm)

3.2 Fantrip on iodine-passivated Au(111)

Step-edges

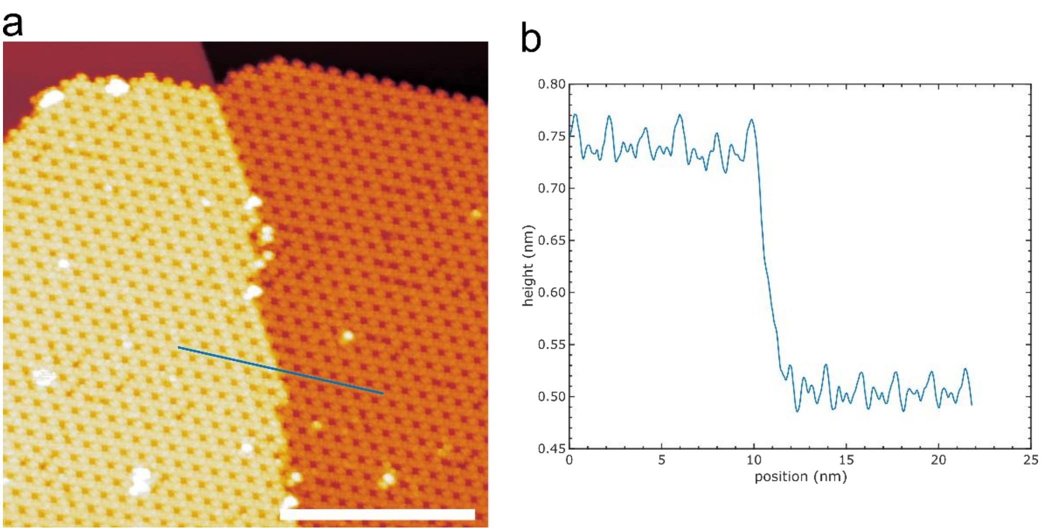

Fig. S3 Fantrip self-assembly on I-Au(111) at step-edges. **a)** STM image of fantrip self-assembly showing a monoatomic step-edge of the surface running in almost vertical direction. The fantrip domains coherently extend over both the lower (darker) and the upper (brighter) terrace, i.e. conform to the same lattice. This indicates structural coherence, suggesting that fantrip can self-assemble across step-edges. **b)** Line-profile along the path indicated by the blue line in a); The height difference of ~0.25 nm, corresponding to a monoatomic Au(111) step-edge, is accommodated directly at the step edge. (tunneling parameters and scale bar: -2.20 V, 10 pA, 20 nm)

Cold deposition

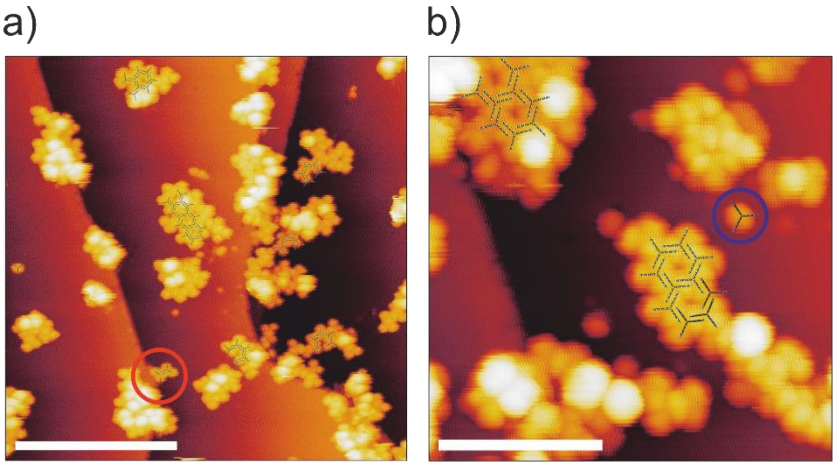

Fig. S4 a) / b) STM images acquired after fantrip deposition onto cooled I-Au(111) held at a temperature of ~80 K. Thereby, fantrip diffusion, and consequently self-assembly were kinetically hindered, resulting in smaller dispersed aggregates of variable size rather than extended domains. We routinely observed dimers (red circle in a), often attached to step-edges) and round isolated features (blue circle in b)). Their sizes are compatible with a single fantrip molecule with its anthracene blades adsorbed edge-on. Although we anticipate smearing out of internal contrast by thermal motion, the assignment is not unambiguous. (tunneling parameters and scale bars: a) +2.22 V; 3 pA, 20 nm; b) +2.15 V; 5 pA, 8 nm)

3.3 Antrip on pristine Au(111)

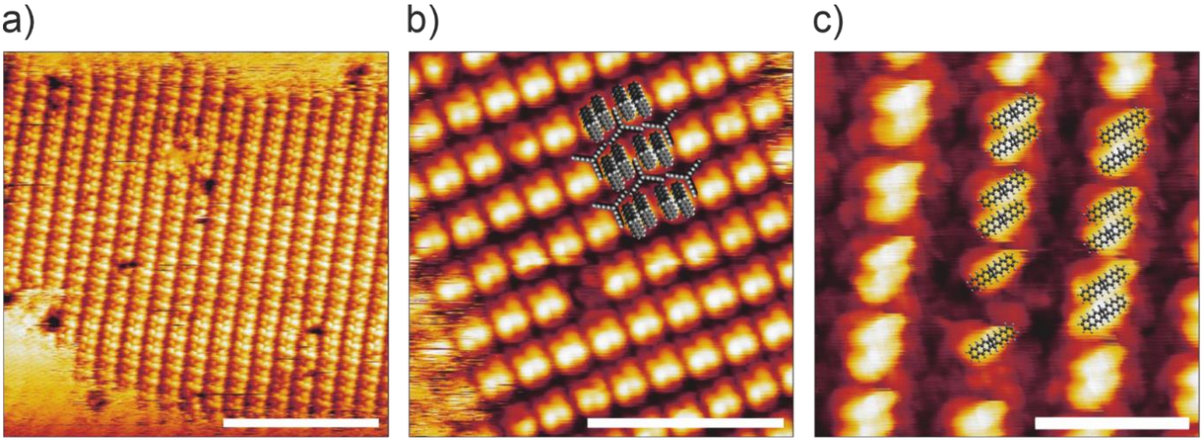

Fig. S5 a) – c) STM images acquired after antrip deposition onto pristine Au(111). Analogous to fantrip, each protrusion corresponds to a single antrip molecule adsorbed with two anthracene blades parallel to Au(111) in accord with the scaled overlay in c). In contrast to fantrip, the monolayer is comprised of antrip dimers that are periodically arranged and separated by small gaps. The difference to fantrip is readily explained by the absence of intermolecular hydrogen bonds, as antrip lacks the fluorine substituents, hence an hydrogen bond acceptor. Therefore, molecule-surface interactions become more important and presumably imprint this irregularity of intermolecular spacings. Moreover, it is also possible to fit antrip molecules with edge-on adsorbed anthracenes into the gaps as shown in b). This offers an alternative explanation for the gaps, where the upright antrip molecules additionally stabilize the structure by π-π interactions in a T-shape configuration. (tunneling parameters and scale bars: a) -1.00 V; 5 pA, 20 nm; b) -1.00 V; 5 pA, 4nm; c) +1.00 V; 5 pA, 4nm)

3.4 Antrip on iodine-passivated Au(111)

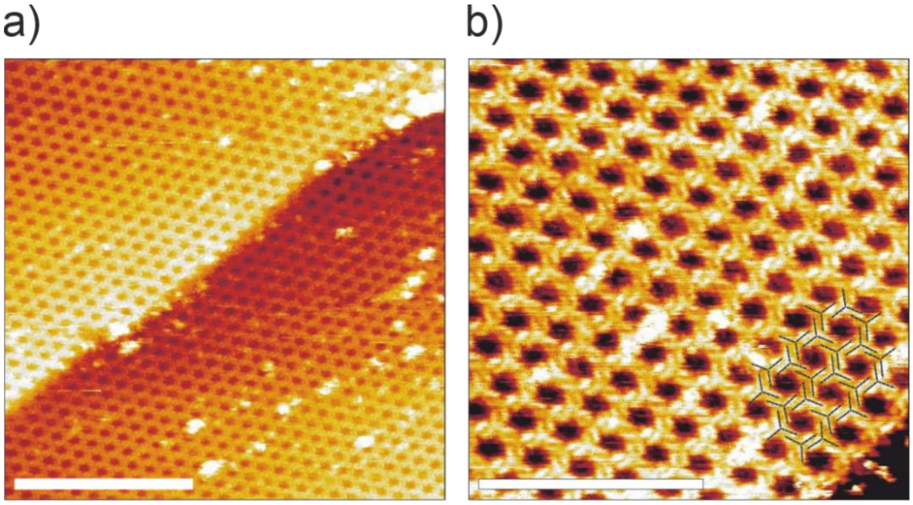

Fig. S6 a) overview and **b)** close-up STM images acquired after antrip deposition onto I-Au(111). Antrip self-assembles into a hexagonal porous monolayer with a lattice parameter of a = b = 1.95 ± 0.10 nm. The antrip and fantrip monolayers on I-Au(111) are isostructural (Fig. 4 of main manuscript). This implies antrip adsorption with all anthracene blades edge-on and face-to-face stacking as indicated by the overlay in the lower right corner of b). Remarkably, the antrip structure on I-Au(111) also adopts the same commensurate 4×4 superstructure (cf. Fig. S2) as fantrip despite notable differences in the optimized lattice parameters of free-standing monolayers (main manuscript, Figure 4f). (tunneling parameters and scale bars: a) +1.10 V, 30 pA, 20 nm; b) +1.10 V; 30 pA, 10 nm)

4 Additional DFT results

4.1 Free-standing monolayers

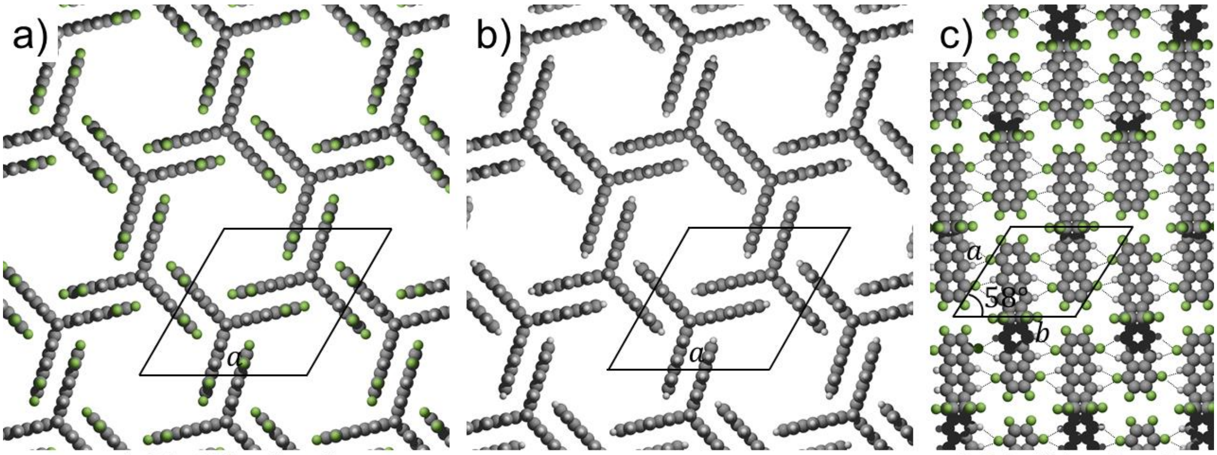

Fig. S7 Estimating the intermolecular binding strength of free-standing monolayers by DFT calculations. Hexagonal monolayers with face-to-face stacked anthracene blades as observed on I-Au(111) for **a)** fantrip (a=b=2.08 nm) and **b)** antrip (a=b=2.01 nm). **c)** Densely packed arrangement of fantrip molecules (a=1.32 nm, b=1.50 nm, γ=58°); The molecular geometry was frozen in the adsorption configuration on pristine Au(111), i.e. with two anthracene blades adsorbed flat on the surface. Respective binding energies per molecule are indicated below. For c) the angle γ was fixed to the experimental value of 58°, while independent optimization of the lattice parameters a and b resulted in values that came out very close to the experiment. The flat adsorption of the anthracene blades facilitates intermolecular hydrogen bonds between fluorine and hydrogen (indicated by dashed lines), giving rise to additional stabilization.

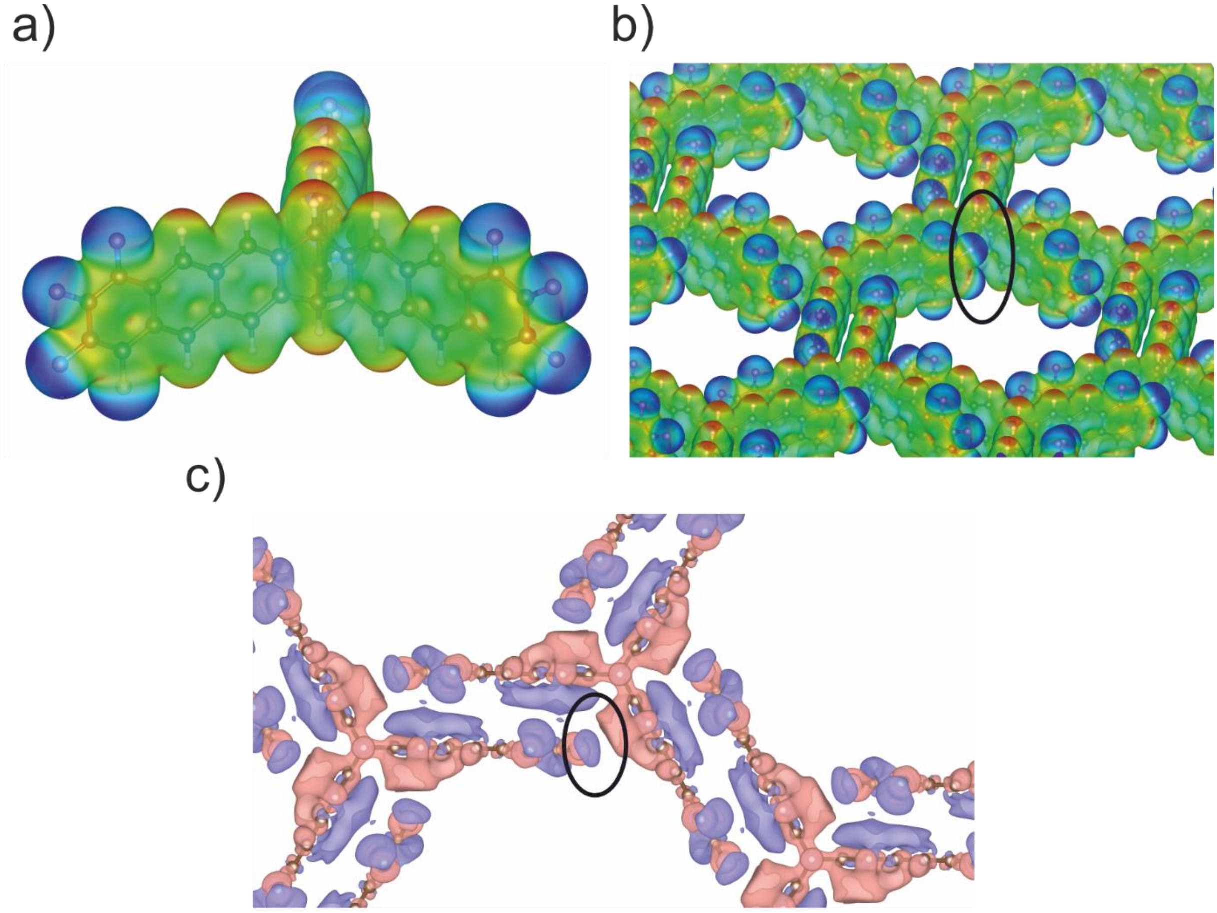

Fig. S 8 Electrostatic interactions in free-standing monolayers. Electrostatic potential maps for **a)** single fantrip molecules in vacuum and **b)** fantrip molecules in the hexagonal free-standing monolayer (blue (red) corresponds to negative (positive) electrostatic potential). As expected, the partial negative charges acquired by the highly electronegative fluorine-substituents give rise to a negative electrostatic potential. The face-to-face stacking in the monolayer packing features an attractive electrostatic interaction with the slightly positive hydrogen-substituents. Yet, the monolayer packing also acts back on fantrip's charge distribution. This becomes evident in **c)** the charge difference plot for monolayer formation with reference to isolated fantrip molecules in vacuum (blue (red) corresponds to electron accumulation (depletion)). The fluorine-substituents acquire more negative charge in the monolayer packing, while electrons are shifted away from the almost perpendicular anthracene blades at the side to which they point. This results in a strengthening of π-π interactions between anthracene blades in the T-shaped configuration (highlighted by black circles in b) and c)).

4.2 Single fantrip molecules on pristine Au(111)

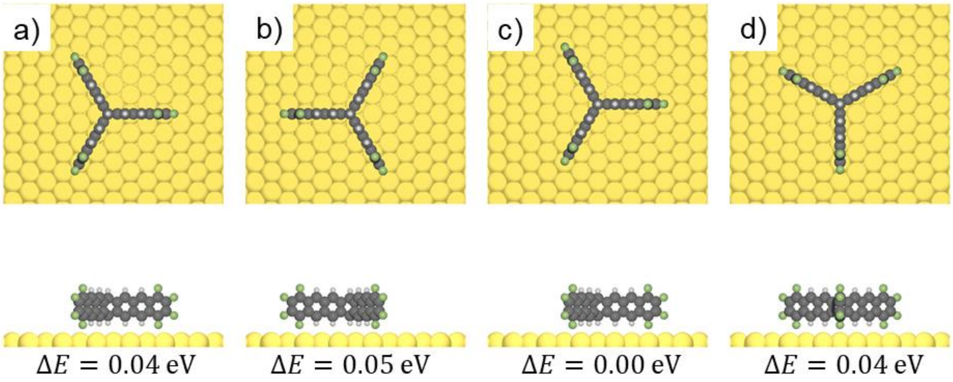

Fig. S9 Adsorption structures and relative energies of single fantrip molecules on pristine Au(111) with all anthracene blades adsorbed edge-on. **a) – d)** final structures obtained after optimization carried out with different start geometries. The energies, which were calculated relative to the most stable adsorption site c), show a low site-variability, suggesting a small diffusion barrier for this hypothetical adsorption geometry.

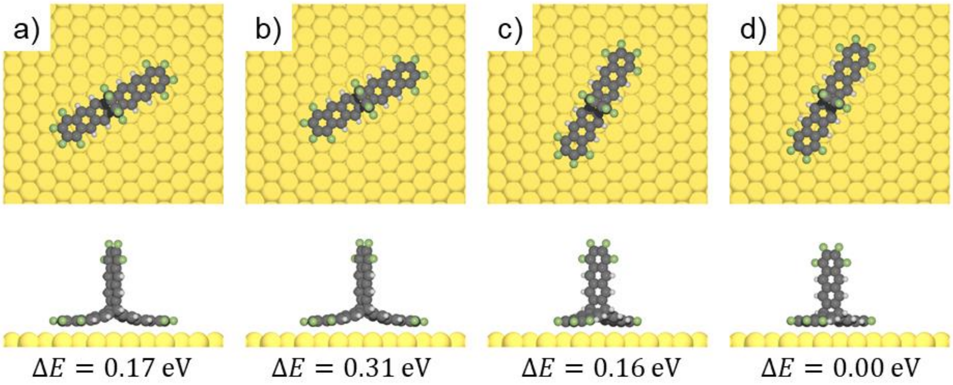

Fig. S10 Adsorption structures and relative energies of single fantrip molecules on pristine Au(111) with two anthracene blades adsorbed parallel and the third upright. **a) – d)** final structures obtained after optimization carried out with different start geometries. The energies are stated with respect to the most stable adsorption configuration d).

4.3 Single fantrip molecules on iodine-passivated Au(111)

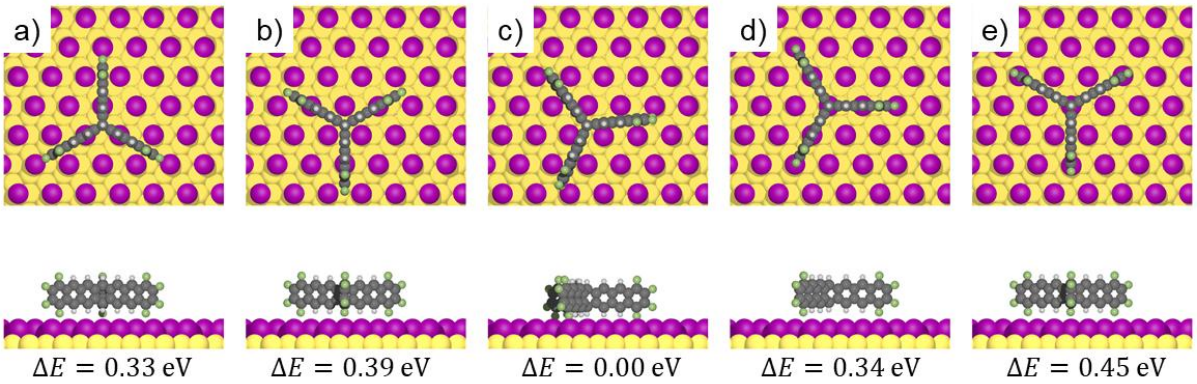

Fig. S11 Adsorption structures and relative energies of single fantrip molecules on iodine-passivated Au(111) with all anthracene blades adsorbed edge-on. **a) – e)** final structures obtained after optimization carried out with different start geometries. The energies are stated with respect to the most stable adsorption configuration c). The site variability of adsorption energies is relatively large. In the clearly favored adsorption geometry in c), the anthracene blades adsorb within the troughs between the iodine atoms, facilitating an energetically favourable closer proximity to the underlying Au(111) surface and iodine monolayer.

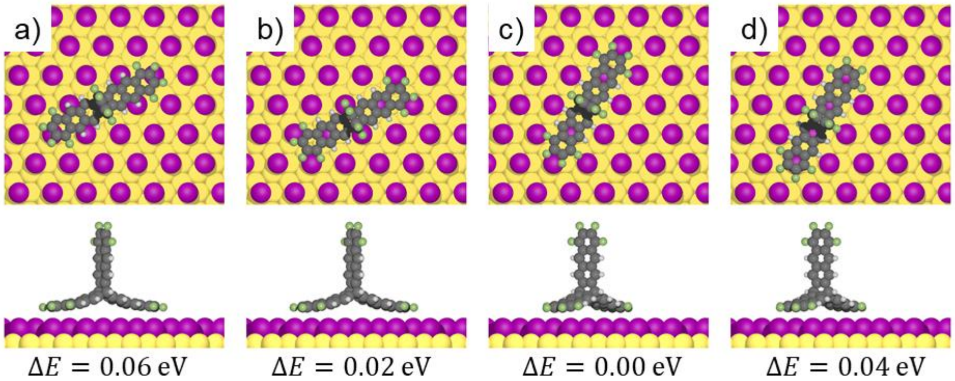

Fig. S12 Adsorption structures and relative energies of single fantrip molecules on iodinated Au(111) with two anthracene blades adsorbed parallel and the third upright. **a) – d)** final structures obtained after optimization carried out with different start geometries. The energies are stated with respect to the most stable adsorption configuration c).

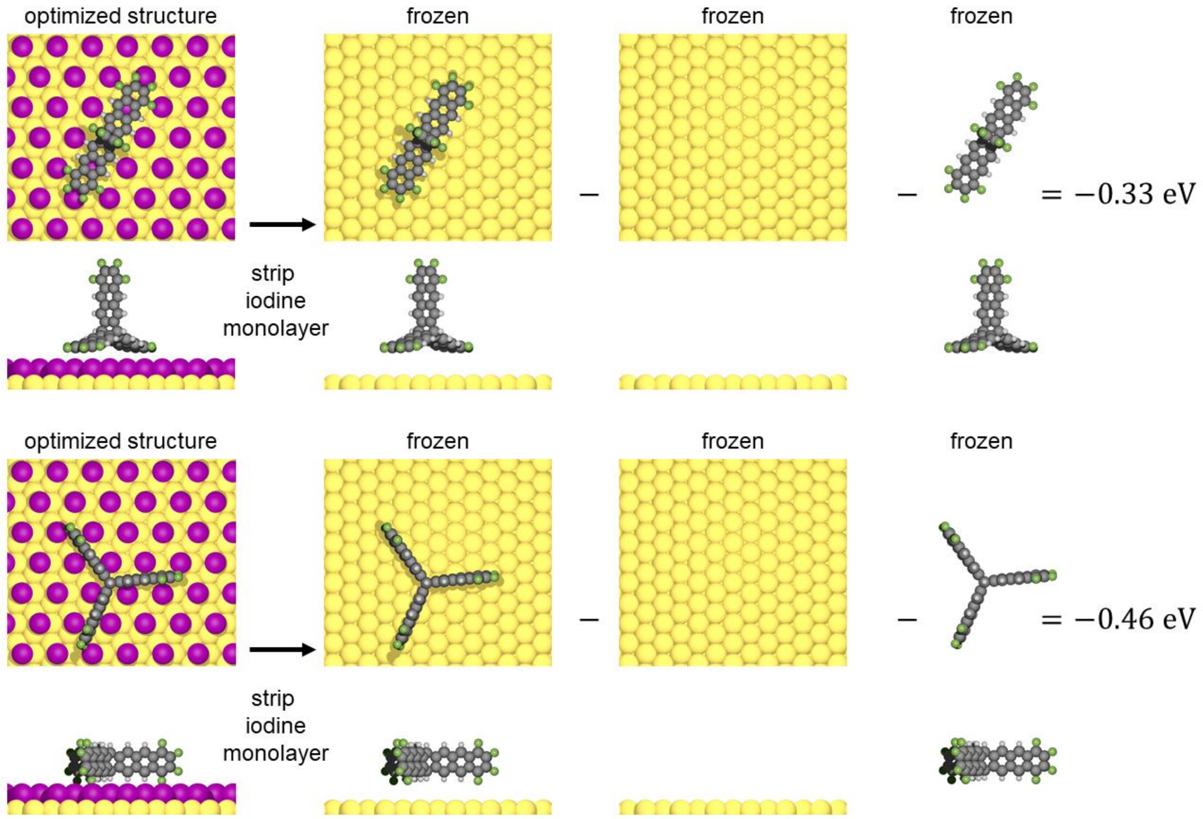

Fig. S13 Scheme to illustrate how the influence of direct interactions with the Au(111) surface was estimated for adsorption on I-Au(111). Optimized structures of single fantrip molecules on I-Au(111) with two anthracene blades adsorbed parallel (upper row) and all anthracene blades adsorbed edge-on (lower row) were used as starting point. Then the iodine monolayer was stripped, while freezing the atomic coordinates of both fantrip and Au(111). Adsorption energies were evaluated in the usual way, i.e. by subtracting energies of the bare Au(111) surface and isolated fantrip molecules (using structures of the adsorbed state). Thereby, we find an approximately 0.13 eV larger energy for the edge-on adsorption geometry. This is rationalized by the lower adsorption height for edge-on adsorption, where fantrip aligns within the troughs of the iodine monolayer.

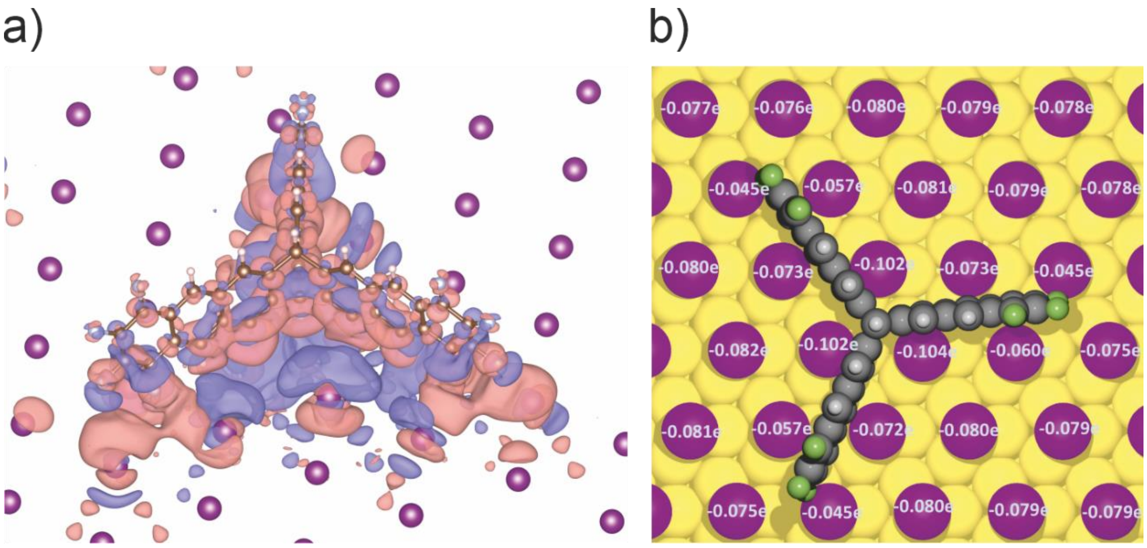

Fig. S14 Charge redistribution for fantrip adsorption on I-Au(111). The calculations are based on the respective lowest energy structure (cf. Fig. S11c), where fantrip adsorbs with its anthracene blades edge-on aligned in the troughs of the iodine monolayer. **a)** Charge difference plot (blue (red) corresponds to electron accumulation (depletion)) for adsorption with reference to isolated molecules in vacuum and an unperturbed I-Au(111) surface, respectively (iodine atoms are depicted as purple spheres, the underlying Au(111) has been omitted for clarity). The plot exemplifies that negative charge is shifted away from the iodine atoms underneath fantrip's highly electronegative fluorine-substituents toward the center of the molecule. **b)** Bader charge analysis of the iodine atoms in the monolayer. For the pristine I-Au(111) surface chemisorbed iodine atoms acquire a minor negative charge of -0.078 e. Evidently, the changes in the proximity of the fluorine-substituents are extremely small. Yet, even the most affected iodine atoms still remain negatively charged, ruling out a favourable electrostatic interaction between fluorine and iodine.

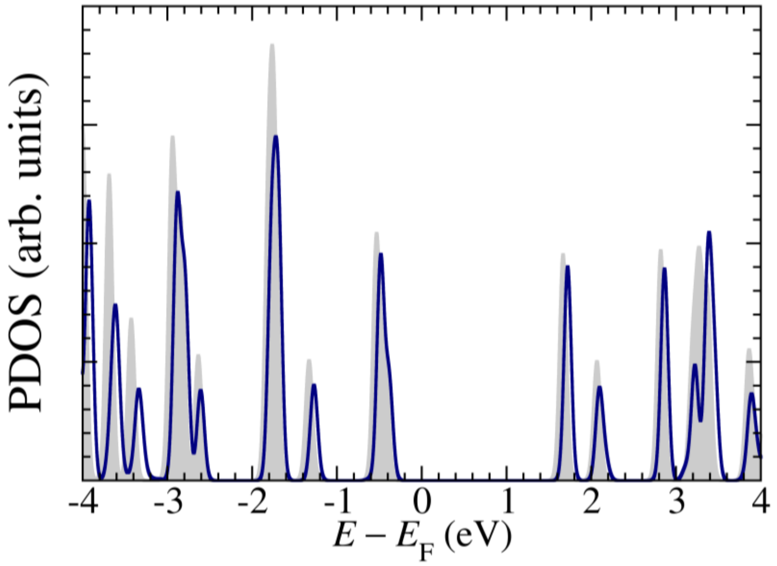

Fig. S15 Partial Density of Electronic States (PDOS) of single fantrip molecules in vacuum (gray filled area) and adsorbed on I-Au(111) in the configuration shown in Fig. S11 (dark blue line). For this comparison, the vacuum levels for both systems have been aligned. The differences remain astonishingly insignificant, clearly indicating that fantrip's electronic structure is not significantly altered upon adsorption. This is an unambiguous signature for physisorption on the weakly interacting I-Au(111) surface.

4.4 STM image simulations

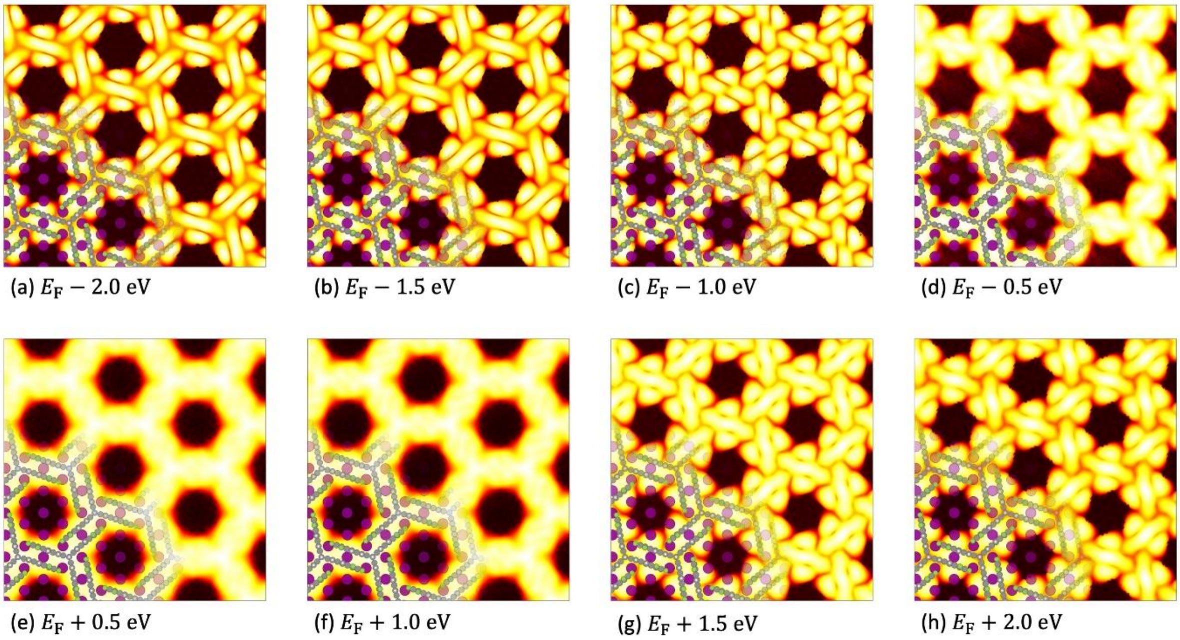

Fig. S16 STM image simulations of a hexagonal fantrip monolayer adsorbed on I-Au(111). The monolayer structure is based on the experimentally observed 4×4 superstructure and the DFT-optimized adsorption site of single fantrip molecules (cf. Fig. S11 c)). Electronic states were evaluated in the energy range between Fermi level (E_F) and **a)** -2.0 eV to **h)** +2.0 eV as indicated. The overlaid structures in the lower left corners are meant to provide orientation.

5 Synthesis of fantrip and antrip

The synthesis and characterization of fantrip is described in detail in the literature.[10] Likewise, the synthesis of antrip was conducted according to the literature,[11] and the structure of the product was confirmed by ^{1}H NMR spectroscopy (cf. Fig. S17). ^{1}H NMR (500 MHz, CDCl3): 5.87 (s, 2H), δ 7.44 – 7.41 (dd, J = 8.05, 4.1 Hz, 6H), 7.98 – 7.95 (dd, J = 8.25, 3.95 Hz, 6H), 8.09 (s, 6H), 8.34 (s, 6H).

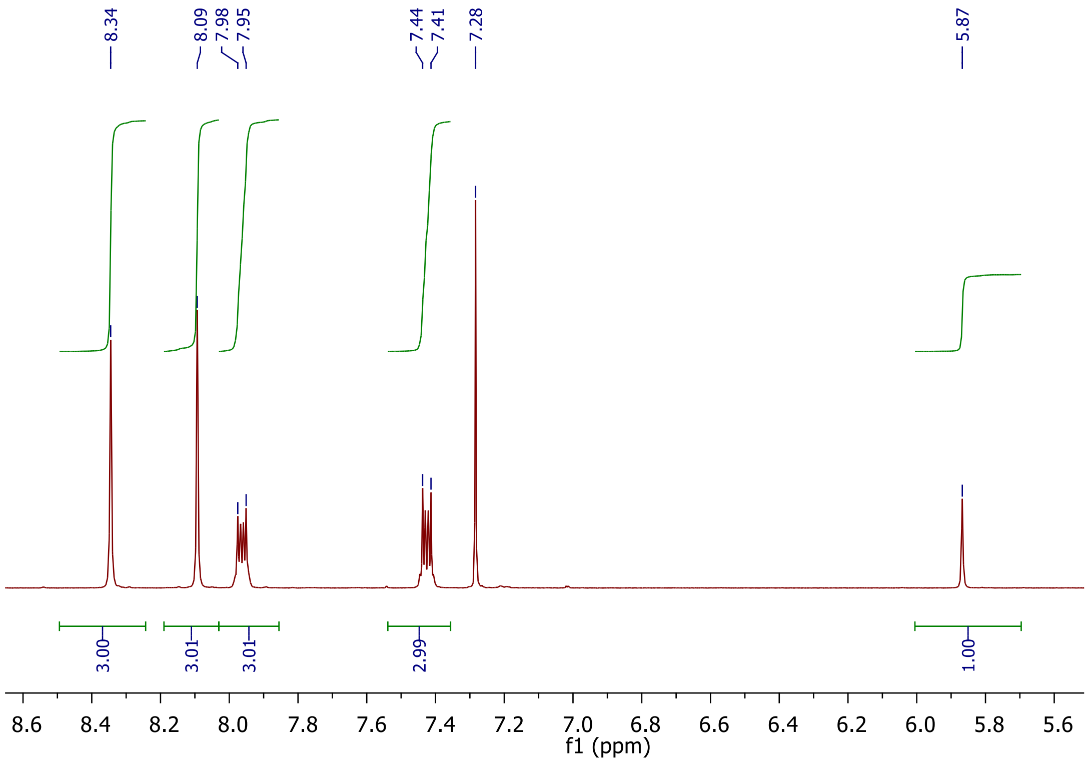

Fig. S17 ^{1}H NMR spectrum of antrip in $CDCl_3$.

6 References

[1] T. Breuer, M. Klues, G. Witte, *J. Electron. Spectrosc.* **2015**, *204*, 102-115.

[2] G. Kresse, J. Furthmüller, *Phys. Rev. B* **1996**, *54*, 11169-11186.

[3] P.E. Blöchl, *Phys. Rev. B* **1994**, *50*, 17953-17979.

[4] a) M. Dion, H. Rydberg, E. Schröder, D.C. Langreth, B.I. Lundqvist, *Phys. Rev. Lett.* **2004**, *92*; b) T. Thonhauser, V.R. Cooper, S. Li, A. Puzder, P. Hyldgaard, D.C. Langreth, *Phys. Rev. B* **2007**, *76*.

[5] I. Hamada, *Phys. Rev. B* **2014**, *89*.

[6] J. Björk, S. Stafstrom, *Chemphyschem* **2014**, *15*, 2851-2858.

[7] Z.V. Zheleva, V.R. Dhanak, G. Held, *Phys. Chem. Chem. Phys.* **2010**, *12*, 10754-10758.

[8] J. Tersoff, D.R. Hamann, *Phys. Rev. Lett.* **1983**, *50*, 1998-2001.

[9] N. Lorente, M. Persson, *Faraday Discuss.* **2000**, *117*, 277-290.

[10] L. Grossmann, B.T. King, S. Reichlmaier, N. Hartmann, J. Rosen, W.M. Heckl, J. Björk, M. Lackinger, *Nat. Chem.* **2021**, *13*, 730-736.

[11] R. Bhola, P. Payamyar, D.J. Murray, B. Kumar, A.J. Teator, M.U. Schmidt, S.M. Hammer, A. Saha, J. Sakamoto, A.D. Schlüter, B.T. King, *J. Am. Chem. Soc.* **2013**, *135*, 14134-14141.
